# A Comprehensive Estimation of the Economic Effects of Meteorological Services Based on the Input-Output Method

**DOI:** 10.1155/2014/904693

**Published:** 2014-01-21

**Authors:** Xianhua Wu, Guo Wei, Lingjuan Yang, Ji Guo, Huaguo Lu, Yunfeng Chen, Jian Sun

**Affiliations:** ^1^Collaborative Innovation Center on Forecast and Evaluation of Meteorological Disasters, Nanjing University of Information Science & Technology, Nanjing, Jiangsu 210044, China; ^2^School of Economics and Management, Nanjing University of Information Science and Technology, Nanjing, Jiangsu 210044, China; ^3^Department of Mathematics & Computer Science, University of North Carolina at Pembroke, Pembroke, NC 28372, USA; ^4^School of Languages and Cultures, Nanjing University of Information Science and Technology, Nanjing, Jiangsu 210044, China; ^5^Popular Science Propaganda Center, China Meteorological Administration, Beijing 100081, China; ^6^Public Meteorological Service Center, China Meteorological Administration, Beijing 100081, China

## Abstract

Concentrating on consuming coefficient, partition coefficient, and Leontief inverse matrix, relevant concepts and algorithms are developed for estimating the impact of meteorological services including the associated (indirect, complete) economic effect. Subsequently, quantitative estimations are particularly obtained for the meteorological services in Jiangxi province by utilizing the input-output method. It is found that the economic effects are noticeably rescued by the preventive strategies developed from both the meteorological information and internal relevance (interdependency) in the industrial economic system. Another finding is that the ratio range of input in the complete economic effect on meteorological services is about 1 : 108.27–1 : 183.06, remarkably different from a previous estimation based on the Delphi method (1 : 30–1 : 51). Particularly, economic effects of meteorological services are higher for nontraditional users of manufacturing, wholesale and retail trades, services sector, tourism and culture, and art and lower for traditional users of agriculture, forestry, livestock, fishery, and construction industries.

## 1. Introduction

The production of sufficient food, fuel, and fiber to meet the world's needs in a sustainable manner relies not only on the natural resources for growing them but also critically upon favorable weather conditions [1]. In recent years, frequent severe weather conditions such as droughts, flood, heavy snowfalls, and high temperatures have increasingly raised governmental and public concerns about meteorological services. However, to promote the utilization of valuable meteorological services, benefits need to be demonstrated quantitatively and answers to relevant questions need to be convincible. Critical questions include those regarding the economic effects, their measures, and estimation methods and models. For example, what are some quantitative results regarding the economic effects saved from disastrous weathers due to accurate meteorological service forecasts? What are some meaningful and reasonable measures for estimating such benefits of meteorological services? Unfortunately, there have been no effective methods and models established in the literature that can be employed by scientists for the desired estimation of the economic effects attributable to meteorological services. One difficulty is to correctly identify the economic effects saved from meteorological services as they are associated with each link of production or consumption. Another difficulty is to determine the costs operating the whole process of meteorological services that requires a wide range of considerations, including weather forecasting systems and assessment of many indirect costs. Still another difficulty is to describe a practical demand function—the demands of meteorological services can be highly implicit at present. Moreover, highly demanded assessment methods and models of economic effects benefited from meteorological services are also related to assessment policies, processes, and practice [2].

Attempts in overcoming these difficulties were proposed by many researchers. Studies and exploratory trials have been carried out for exploring effective technologies and solutions both in China and abroad. These solutions can be roughly classified into three categories: direct field investigation, expert knowledge and qualitative analysis, and input-output assessment. Among the methods in the direct field investigation category, the focus is to measure the effects on the service objects that are rescued due to the use of meteorological services for users through the direct field investigation. Nguyen et al. calculated the economic effects of typhoon warning services in Vietnam [3]. By designing questionnaires based on the contingent valuation method, Birol et al. estimated the economic effect of water resources management [4]. Within the second category, expert knowledge and qualitative analysis, one calculates the effect brought by meteorological services using expert knowledge, Delphi method, or alike and then combining qualitative analysis measures and quantitative analysis results. Krieger and Green put forward the decision and optimization model of service effect estimation [5]. Recently, Xu estimated meteorological service effects using the Delphi method [6]. Integrating the experts' knowledge into the epidemiology-based exposure-response functions, Kan and Chen assessed the health based economic cost of particulate air pollution in urban areas of Shanghai of China [7]. For the third category, input-output assessment, researchers compute the associated effects brought by services using an input-output method. Chen and Yin proposed a computing method of indirect and complete economic effects [8]. Using input occupancy output techniques, Wang established an accounting method for estimating the complete forward and backward economic effects for all industries in the national economy [9]. Hewings and Sonis proposed some concepts and algorithms relating to forward and backward linkage, correlative relations within industries, and output multipliers in input-output analysis [10]. Recently, Chen et al. estimated the associated social economic effects brought by oyster breeding in Taiwan [11], and Kerschner and Hubacek assessed the potential economic effects of peak oil using the input-output analysis [12]. These are just some of the existing researches achieved so far.

The Delphi method (a structured communication technique, originally developed as a systematic, interactive forecasting method which relies on a panel of experts), is frequently employed by researchers. As a subjective, qualitative method, the Delphi method is essentially a feedback anonymous letter of inquiry method, with several advantages such as full role of experts, brainstorming, and high accuracy. However, it should be noticed that this expert opinion method can be utilized only when there is a lack of sufficient information—due to its drawbacks including (1) no clear criteria regarding expert selection, (2) lack of rigorous scientific analysis of the results, and (3) the final convergence of views having a tendency to follow the crowd.

Moreover, except for a few case studies, most researchers only estimated the direct effects without investigating the indirect effects, which results in a gap in the literature of meteorological services. Moreover, most researchers simply combined field investigations and expert knowledge for estimating service effects in a single region, industry or enterprise; few studies utilized the input-output method to estimate the comprehensive service effects. Research, using input-output methods to calculate the associated and indirect economic effects, is rare. Chen and Yin and Wang, respectively, defined the concepts of indirect economic effect and complete economic effect and put forward corresponding algorithms [8, 9].

Centered about consuming coefficients, partition coefficients and Leontief inverse matrix, we present in this paper the concepts of associated economic effect, indirect economic effect, and complete economic effect, input-output method, and algorithms that estimate, respectively, the associated, indirect, and complete economic effects. Generally, the direct economic effect is assumed to be known. In addition, illustrative examples are provided to explain the concepts and demonstrate the reliability and practicability of the models and algorithms regarding the theoretical innovation and economic effects for applications.

Specifically, since the input-output table reflects more accurately the technical and economic relationships between industries in the national economy [13], it has become an ideal tool for calculating the associated and indirect economic effects of industries. According to the principle of doing certain things and refraining from doing other things, we present assessment models for associated, indirect, and complete economic effects based on the input-output table. Taking the meteorological service data in Jiangxi province in 2007 as an example, we obtained a series of results regarding the economic effects of meteorological services.

Section 2 describes the concepts, principles, and hypotheses.  Section 3 introduces our estimation methods and algorithms.  Section 4 illustrates our methods and algorithm with an actual example. The last section, Section 5, lays out the concluding remarks.

## 2. Concepts, Principles, and Hypotheses

In this section we will define concepts that describe economic effects and then introduce the principle of input-output table and several hypotheses.

### 2.1. Definition of Concepts

Here we assume that the direct meteorological service object is industry *i*.

The direct economic effect of meteorological services is the increased economic effect due to the use of these meteorological services. To further explain this concept, assume that two similar industries, one utilizing the meteorological services and the other not, have similar input levels but with different outputs. The difference between the outputs that is beneficial from the use of the meteorological services is referred to as the direct economic effect.

The associated economic effect of industry *i* is the economic effect due to the demands of other industries for the products (or services) produced (or provided) by industry *i*, which is to be calculated through the interdependency coefficients.

The indirect economic effect of industry *i* is the sum of economic effects for industry *i* and other industries generated indirectly through an economic and technological relation.

The complete economic effect of industry *i* is the increment of final output in all industries of national economy system brought by direct economic effect with the circulation of production-consumption.

### 2.2. Principle of the Input-Output Table

The input-output table describes resources of inputs and usages of outputs on all industries of the national economy in a matrix form for a period of time (usually one year). It reveals the quantitative relations which are not only interdependency but also mutually restraining for all industries of the national economy. As an important part of the national economic accounting system, the input-output table of China consists of three parts named as Quadrants I, II, and III.  Table 1 is illustrated.

Interconnected, these three parts of input-output table fully and systematically reflect the interrelations of all industries of the national economy, during the production cycle process from production to usage, by view of total quantity and structure of input-output. The following are some basic balance relationships in the input-output table:

(i) line balance:

(1) Intermediate  use+Final  use+Others    =Total  output+Inflow,

(ii) column balance:

(2) $$\begin{array}{c} \text{Intermediate}\;\text{input}+\;\text{Increment}\;=\;\text{Total}\;\text{input}, \end{array}$$

(iii) gross balance:

(3) Total  input=Total  outputTotal  input  in  certain  industry  =Total  output  in  this  industryTotal  intermediate  use=Total  Intermediate  input,

(iv) interdependency between industries.

The interdependency between industries can be expressed as follows:

(4) $$\begin{array}{c} x_{i}=\underset{j}{\sum }(a_{ij}x_{j}+c_{j}),\;i=1,2,\ldots ,n, \end{array}$$

or in the matrix form *X* = *AX* + *C*, where *x*_*i*_ is the total output of industry *i*, *c*_*i*_ is the final demand of industry *i*, and *a*_*ij*_ is the ratio of the input of industry *i* over the total demand of industry *j*  (1 ≤ *i*, *j* ≤ *n*). Given *n* industries, *a*_*ij*_ characterizes the distribution of inputs contributed by the *n* industries (*i* = 1,2,…, *n*) to the total input required by industry *j* [15]. Matrix *A* will be called the technical coefficient matrix.

### 2.3. Hypotheses of Input-Output Model

The input-output model is a simplification of the Walrasian general equilibrium model [16]. There are three main hypotheses.

*H*_1_ : Pure industry: assume that each industry only produces one kind of product with one production technology. Meanwhile no different production technology can be selected or mutually replaced in the process of production across different industries.

Models holding *H*_1_ can reflect the composition of material consumption and the relation between production and technology more accurately.

*H*_2_ : Each technical coefficient is relatively fixed. Regardless of factors of technical progress and increase of labor productivity, assume that each direct consumption coefficient (i.e., technical coefficient) *a*_*ij*_ is fixed in a given period, that is to say, ignore influences of relevant dynamic factors.

Here, dynamic factors include change of time, technology, price, industry, or product structure among others. With *H*_2_, the analysis will be much simplified.

*H*_3_ : Linear relation: assume that there is a positive and proportional relationship between input and output in all industries of the national economy.

*H*_3_ is closely related to *H*_2_. On the premise that direct consumption coefficient *a*_*ij*_ is fixed, there must be a positive and proportional relationship between consumption and production, fixed consumption in production ignored.

Despite exceeding the bounds of reasonable stipulations, these three hypotheses, presented in the forms subject to some ideal conditions, are fundamental in describing more general economic production relations and expanding the scope of input-output method, thus helpful for other researches as well.

## 3. Estimation Models of Associated, Indirect, and Complete Economic Effects

### 3.1. Estimation Models of Associated Economic Effects

*(1) Direct Interdependency*. The direct interdependency means the economic and technological relation between an industry and another industry which needs products or services from the former. It is usually measured by the direct distribution coefficient *h*_*ij*_  (*i*, *j* = 1,2,…, *n*). *h*_*ij*_ represents the proportion of the products or services directly used as intermediate products distributed from industry *i* to industry *j* in the total output. The formula calculating *h*_*ij*_ is

(5) $$\begin{array}{c} h_{ij}=\frac{x_{ij}}{X_{i}},\;\left(i=1,2,\ldots ,n,\;\;j=1,2,\ldots ,n\right), \end{array}$$

where *x*_*ij*_ denotes the products or services used as intermediate products distributed from industry *i* to the latter and *X*_*i*_ is the total output of industry *i*.

Clearly, the higher the direct distribution coefficient of an industry to another, the greater the direct interdependency of the former industry for the latter industry, and the more obvious direct driving effect.

*(2) Complete Distribution Coefficient*. The complete distribution coefficient *d*_*ij*_  (*i*, *j* = 1,2,…, *n*) is the complete distribution of industry *j* provided per unit of value-added in industry *i*. It can be calculated on the basis of complete consumption coefficient. The matrix form formula calculating *d*_*ij*_ is given by

(6) $$\begin{array}{c} D={\left(I-H\right)}^{-1}-I, \end{array}$$

where *I* is the *n* by *n* identity matrix and *H* is the matrix of direct distribution coefficients, that is, *H* = (*h*_*ij*_). (*I*−*H*)^−1^ describes the total accumulative distribution effect and it is similar to the Leontief inverse matrix (*I*−*A*)^−1^.

The bigger the complete distribution coefficient, the greater the motivational effect of complete supply, and the bigger the complete interdependency between industries. Complete distribution coefficients not only reflect the direct impact among industries but also reflect the indirect impact of every level, which is more comprehensive for analyzing relativity between industries.

*(3) Associated Economic Effect*. The associated contribution is the added value created by the service effect of an industry for the production of intermediate input. According to the balance relation of the input-output table and the theory of industry interdependency, the associated contribution of industry *i* for the national economy is

(7) $$\begin{array}{c} E_{i}=\overset{}{\underset{}{\sum }}d_{ij}Y_{ij}. \end{array}$$

Here, *d*_*ij*_ is the complete distribution coefficient of industry *i* for products of industry *j*, *Y*_*ij*_ is the direct economic effect of meteorological service for industry *j* due to industry *i*, and *E*_*i*_ in (7) is called the associated economic effect of meteorological service from industry *i*.

### 3.2. Estimation Model of Indirect Economic Effect

Recall, from Section 2.1, that the indirect economic effect is the economic effect brought indirectly through an economic and technological relation. According to the input-output model in Table 1, we can get the total output of one industry as follows:

(8) $$\begin{array}{c} \overset{n}{\underset{j=1}{\sum }}X_{ij}+Y_{i}=X_{i},\;\left(i=1,2,\ldots ,n\right). \end{array}$$

Here, *X*_*ij*_ is the intermediate consumption, representing the product value consumed in industry *j* which is provided by industry *i*, *Y*_*i*_ is the product value which is used as final use in industry *i*, and *X*_*i*_ is the total output of industry *i*. By adding the direct consumption coefficients *a*_*ij*_ = *x*_*ij*_/*X*_*j*_ into the model, (8) can be turned into

(9) $$\begin{array}{c} \overset{n}{\underset{j=1}{\sum }}a_{ij}X_{j}+Y_{i}=X_{i},\;\left(i=1,2,\ldots ,n\right). \end{array}$$

The matrix form of (9) is *AX* + *Y* = *X*, where *A* is the technical coefficient matrix, and we have

(10) $$\begin{array}{c} X={\left(1-A\right)}^{-1}Y. \end{array}$$

In the input-output table, service effects can be represented as the increase of final output, assuming that the final outputs of other industries are fixed. On the basis of the direct economic effect of a certain industry produced by services, we will explain the indirect effect as Δ*X* brought by the direct economic effect of an industry. Accordingly, (10) changes into the incremental form:

(11) $$\begin{array}{c} \Delta X={\left(I-A\right)}^{-1}\Delta Y. \end{array}$$

Here, Δ*X* is the indirect effect of this industry brought by meteorological services, (*I*−*A*)^−1^ is the Leontief inverse matrix, and Δ*Y* is the direct effect of this industry brought by services which is represented as the increase of final use.

### 3.3. Estimation Model of Complete Economic Effect

According to the previously established estimation model for the indirect economic effect, the increment of final use brought by the direct effect in the first round is Δ*X* = (*I*−*A*)^−1^Δ*Y*.

The increase of output in the first round will improve resident income, thus increasing resident consumption, which will further lead to the increase of output in the second round. The whole process repeats itself in circle. Of course, there are some hidden assumptions. First, the economic system has an enormous amount of idle productive capacity so that there will not be induced investment in the process of circulation. Second, with the increasing resident income, the marginal propensity to consume is constant. Third, consumption structure does not change as consumption scale changes.

Assume that *α* = (*α*_1_, *α*_2_,…, *α*_*n*_) denotes the vector of resident income structure in each industry (*α*_*i*_ is the ratio of laborers' remuneration in total output for industry *i*). Let *c* denote the marginal propensity to consume. Then *cα* · (*I* − *A*)^−1^Δ*Y* represents the increment for consumption brought by service effect in the first round. Let *w* be the column vector of resident consumption structure coefficient in input-output table. Element *w*_*i*_ in *w* is the ratio of consumption of each industry in the total value of that column. So *w* *cα*(*I*−*A*)^−1^Δ*Y* is the resident consumption increment produced by the increase of final use in the first round. (*I*−*A*)^−1^ *w* *cα*(*I*−*A*)^−1^Δ*Y* is the output increment induced by the increase of final use in the initial state and first round, and so on. This production-consumption-production cycle will go on until the system reaches a new equilibrium. The foregoing is expressed by a mathematical equation as follows:

(12) ΔX=(I−A)−1ΔY+(I−A)−1wcα(I−A)−1ΔY+(I−A)−1wcα(I−A)−1wcα(I−A)−1ΔY+⋯

Then we can get

(13) $$\begin{array}{c} \Delta X={\left(I-A\right)}^{-1}{\left[I-wc\alpha {\left(I-A\right)}^{-1}\right]}^{-1}\Delta Y. \end{array}$$

## 4. The Empirical Analysis of Meteorological Service Effects in Jiangxi Province

In this section, we will utilize the data obtained from Jiangxi province in 2007 to illustrate the concepts and models introduced in this paper (Section 3). We employ the input-output method to estimate the economic effects of meteorological services based on the data given in the 2007 input-output table of Jiangxi [17].

### 4.1. Sample and Data

Zou et al. conducted a research on the evaluation reports of meteorological services in Jiangxi from 2003 to 2007 using the traditional expert investigation method (Delphi method) [14], while our purpose is, by utilizing the input-output model for the data supplied with the 2007 input-output table of Jiangxi, to estimate quantitatively the economic effects rescued from the preventive strategies that are established based on the meteorological information and the interdependency between industrial economic systems. Our setting of the study is different from that of Zou et al. as outlined below.

*(1) Data of Input-Output*. They are from “Input-output table of Jiangxi in 2007” and “Input-output table of China in 2007,” where the latter was compiled by the Economic Accounting Department of the National Bureau of Statistics of China in 2007. The table involves 2 branch classifications; one is a table of 42 industries and, the other, a table of 135 industries. In this paper we use the data of 135 industries. According to the research of Gu et al., the resident marginal propensity to consume *c* in 2007 was 0.66 [18].

*(2) Data on the Direct Economic Effect of Meteorological Services*. Using the expert investigation method, Zou et al. estimated the meteorological service effect on agriculture, forestry, husbandry, and fishery, transportation, construction, production, and supply of electric power, gas, and water, and insurance, manufacturing, resident service, and other services, wholesale and retail sale in Jiangxi province [14]. To correspond to the names of 135 industries in input-output table, we process the same data but utilizing our comprehensive economic effect estimation method as follows.

- Zou, Lu, and Dong synthesized agriculture, forestry, husbandry, and fishery into one industry. In this paper we split it equally into five shares with the same average proportions of 3.61%.
- In Zou, Lu, and Dong's study, there was only an average proportion of direct service effect for general “manufacturing”. In this paper we use 54 kinds of manufacturing in the table of 135 industries with the same proportions of 0.512%.
- In Zou, Lu, and Dong's study, there was only an average proportion of “insurance.” Considering that “insurance” is a subkey of “finance” in the table of 135 industries, we equate “insurance” with “finance,” whose ratio of meteorological service effect in total output is 1.943%.
- In Zou, Lu, and Dong's study, there was only an average proportion of “transportation and warehousing.” In this paper we treat 8 subkeys of “transportation and warehousing” in the table of 135 industries whose proportions are all 2.454% equally.
- In Zou, Lu, and Dong's study, there was only a contribution rate of “resident service and other services.” According to the table of 135 industries, “resident service and other services” will be subdivided into “resident service” and “other services,” whose proportions are all 0.484%.

Specific results are provided in Table 2.

It should be pointed out that the Delphi method is a structured communication technique, originally developed as a systematic, interactive forecasting method which relies on a panel of experts and thus subject to and dependent on the knowledge of the participating experts. Hence, this method is more subjective in nature.

Moreover, since the available meteorological service data sets for Jiangxi province from 2003 to 2007 are quite rich, the input-output method would be more appropriate. In fact, the Delphi (expert opinion) method is utilized only when there is a lack of sufficient information (due to its three drawbacks stated in Section 1).

In contrast, our estimation model (input-output method) for obtaining quantitative results regarding the comprehensive economic effects of meteorological services is established on the inhesion relevance among different industries, which is more objective and provides a practical setting for preventive strategies and other recommendations to rescue in the face of extreme weather conditions.

Specifically, since the input-output table reflects more accurately the technical and economic relationships between industries in the national economy [13], it has become an ideal tool for calculating the associated and indirect economic effects of industries. According to the principle of doing certain things and refraining from doing other things, we present assessment models for associated, indirect, and complete economic effects based on the input-output table. Taking the meteorological service data in Jiangxi province in 2007 as an example, we obtained a series of results regarding the economic effects of meteorological services.

### 4.2. Results and Analysis

By utilizing the estimation equations of associated economic effect (7), indirect economic effect (11), and complete economic effect (13), we summarize the results in Table 3. 

An analysis of Table 3 reaches the following conclusions.

(1) Due to the technical and economic relations between industries, the direct economic effect of services can bring to all industries the associated, indirect, and complete economic effects. The direct economic effect of meteorological service in Jiangxi province in 2007 was 13882.63853 million RMB, which brought the associated economic effect of 39521.90571 RMB, indirect economic effect of 33991.28942 RMB, and complete economic effect of 50105.33474 RMB. Three economic effects, respectively, increased by 1.847-fold, 1.448-fold, and 2.609-fold.

The ratio range of input in associated economic effect in Jiangxi province is about 1 : 85.41–1 : 145.197, the ratio range of input in indirect economic effect is about 1 : 73.44–1 : 124.848, and the ratio range of input in complete economic effect is about 1 : 108.27–1 : 183.059, which are remarkably different from a previous estimation based on the Delphi method (stated below).

Ren studied the “Input-output table of China in 2007” for the whole country using the traditional Delphi method and concluded that the effect ratio range of input in output brought by meteorological services in China is 1:30–1:51 [19].

In contrast, our results suggest that the associated, indirect and complete economic effects brought by meteorological services are so huge that more attention should be paid to this field.

(2) As observed from the calculation of associated economic effect brought by direct economic effect, the top five industries, in order of decreasing proportions of associated effect are refractory product manufacturing (1405.538-fold), special machinery for agriculture, forestry, husbandry, and fishery manufacturing (158.467-fold), cement and gypsum product manufacturing (157.029-fold), brick, stone, and other building material manufacturing (154.352-fold), and ceramic product manufacturing (108.871-fold). The bottom five are construction (0.002-fold), other food manufacturing (0.030-fold), fishery (0.042-fold), animal husbandry (0.083-fold), and agriculture (0.144-fold).

Also, from the calculation of indirect economic effect brought by direct economic effect, the top 5 industries in order of decreasing increments are railway transport equipment manufacturing (15.316-fold), fertilizer manufacturing (8.698-fold), special machinery for agriculture, forestry, husbandry, and fishery manufacturing (8.487-fold), pesticide manufacturing (7.896-fold), and basic chemical raw material manufacturing (5.438-fold). The bottom five construction (0.035-fold), convenience food manufacturing (0.053-fold), fishery (0.060-fold), other food manufacturing (0.090-fold), and animal husbandry (0.148-fold).

Furthermore, as observed from the calculation of complete economic effect brought by direct economic effect, the top 5 industries in order of decreasing increments are railway transport equipment manufacturing (21.810-fold), fertilizer manufacturing (12.1651-fold), special machinery for agriculture, forestry, husbandry, and fishery manufacturing (10.654-fold), pesticide manufacturing (10.602-fold), and wholesale and retail sale (9.113-fold). The bottom five are construction (0.069), fishery (0.231), warehousing (0.522-fold), service of agriculture, forestry, husbandry, and fishery (0.546-fold), and animal husbandry (0.560-fold).

Thus, increments of indirect and complete economic effects on traditional meteorological service objects such as construction, agriculture, forestry, husbandry, fishery, and warehousing are comparatively low, while some industries such as railway transport, equipment manufacturing, and fertilizer manufacturing can reach more indirect and complete economic effects. Consequently, in the future, meteorological service should tilt toward industries including railway transport, equipment manufacturing, and special machinery for agriculture, forestry, husbandry, and fishery manufacturing and provide more targeted fine services to improve their indirect and complete economic effects of meteorological service.

(3) As seen from the calculation of complete economic effect brought by indirect economic effect, the top five industries in order of decreasing increments are culture and art (577.222-fold), tobacco processing (67.673-fold), tourism (26.218-fold), weaving, dyeing, and finishing (16.627-fold), and public facilities management (14.027-fold). The bottom six are construction (0.033-fold), cement and gypsum product manufacturing (0.078-fold), service of agriculture, forestry, husbandry, and fishery (0.092-fold), refractory product manufacturing (0.122-fold), steel rolling processing (0.157-fold), and ferrous metal ore mining (0.157-fold). It indicates that owing to the production-consumption-production cyclic effect, the output multipliers of industries like culture and art, tobacco processing, and tourism are bigger than those of construction, agriculture, forestry, husbandry, fishery, and so on. The results are of course affected by multiple factors such as technical and economic relation, marginal propensity to consume, ratio of resident income in total output, and so on. Because of the increasing relevance of industrial economic system, rapid development of public administration, social organization, education, technology, and the gradual reveal of effect on the policies of expanding domestic demand, the indirect and complete economic effects brought by meteorological service will continue to increase.

While Zou, Lu, and Dong estimated the ratio of meteorological services effect (input-output ratio) for various industries, they did not calculate the economic effects [14]. As a result, we could not make a comparison between our results and theirs.

## 5. Concluding Remarks 

In this paper we introduced the concepts of direct (associated, indirect, and complete) economic effect. Focusing our research on the direct consuming coefficient, the complete consuming coefficient, and Leontief inverse matrix, we have developed estimation methods for the associated, indirect, and complete economic effects. Using the meteorological services in Jiangxi province as a demonstrative example, the concepts and methods have been validated.

The main findings include the following.

1. Higher interdependency between economic systems of different industries exist, implying that the associated, indirect, and complete economic effects brought on by meteorological services could be much larger. For example, the ratio range of input in complete economic effect on meteorological services in Jiangxi province is about 1 : 108.27–1 : 183.059, which is larger than that of previous estimation using Delphi method (1 : 30–1:51), suggesting that the society as a whole should pay more attention to meteorological services.
2. Some industries with higher industrial connection ratios (Leontief inverse matrix), such as steel rolling processing, other nonmetal ore mining, and manufacturing, can achieve more associated, indirect, and complete economic effects. It follows that more attention should be paid to the meteorological services of these industries.
3. Increments of complete economic effect for some industries are relatively larger. These include railway transport, equipment manufacturing, fertilizer manufacturing, special agriculture machinery manufacturing, forestry, livestock, and fishery manufacturing, pesticide manufacturing, wholesale and retail sales, and other services. Consequently, governments should engage meteorological services in these areas.
4. Increments of indirect and complete economic effects on meteorological services traditionally directed to industries like construction, agriculture, forestry, livestock, and fishery, and warehousing are low. In the future, meteorological service providers should both expand their customer range and improve their quality of service in order to ultimately increase their comprehensive economic effects in these domains.

The algorithms developed in this paper are characterized by the following strengths.

1. When used in estimating the economic effects of meteorological services, the algorithms make it possible to fully consider the intrinsic relationship between industries; calculated comprehensive values are more scientific and credible.
2. The algorithms developed can rank the industries with the biggest increments and pick out highly sensitive industries. The results can provide references for development strategy and meteorological service investment decisions.
3. The algorithms can also be used for loss from weather events, for example, for the associated, indirect, and complete economic loss of industrial economic systems caused by disasters or sudden crises (e.g., the impact estimation of “911” event on US aviation). Similarly, the impact estimation for different carbon reduction policies on industrial economic systems, goal programming, and design of industrial regulations, all lend themselves to the use of the algorithms.

Finally, there are still some concerns regarding the concepts, algorithms, and applications of associated, indirect, and complete economic effect estimation mentioned. In particular, the assumption that the relevance within industries is linear, rigid, and static may not reflect exactly reality of dynamic and complex industrial economic systems. To overcome such constraints assumed in the input-output method remains a major focus of future research.

## Figures and Tables

**Table 1:** Basic input-output table of China.

	Output																
	Intermediate use				Final use										Inflow	Others	Total output
Input	Farming forestry, animal husbandry, and fishery	*⋯*	Public administration and social organization	Total intermediate use	Final consumption					Gross capital formation			Total	Total final use
					Resident consumption			Government consumption	Total	Fixed capital formation	Increase in inventory	Total		
					Rural resident consumption	Urban resident consumption	Subtotal							
*Intermediate input *																	
Farming forestry, animal husbandry, and fishery ⋮ Public administration and social organization	Quadrant I				Quadrant II												
Total intermediate input																	

*Increment *																	
Laborers remuneration Taxes on production Depreciation of fixes assets Operating surplus Total increment	Quadrant III																

Total input																	

**Table 2:** Contribution rate of weather service in highly sensitive industries in Jiangxi province, China (Measuring unit: %).

Industry	Direct service effect in 2003–2007	Contribution rate of meteorological service effect
Agriculture	4.07–3.15	3.61
Forestry	4.07–3.15	3.61
Animal husbandry	4.07–3.15	3.61
Fishery	4.07–3.15	3.61
Agriculture, forestry, husbandry, and fishery	4.07–3.15	3.61
Convenience food manufacturing	0.631–0.392	0.512
Milk and dairy manufacturing	0.631–0.392	0.512
Condiments and fermentation products manufacturing	0.631–0.392	0.512
Other food manufacturing	0.631–0.392	0.512
Alcohol and wine manufacturing	0.631–0.392	0.512
Textile product manufacturing	0.631–0.392	0.512
Knitwear, weaving, and its product manufacturing	0.631–0.392	0.512
Textile wearing apparel, footware, and cap manufacturing	0.631–0.392	0.512
Furniture manufacturing	0.631–0.392	0.512
Sport, cultural, and educational supply manufacturing	0.631–0.392	0.512
Basic chemical raw material manufacturing	0.631–0.392	0.512
Fertilizer manufacturing	0.631–0.392	0.512
Pesticide manufacturing	0.631–0.392	0.512
Coating, printing ink, paint, and similar product manufacturing	0.631–0.392	0.512
Synthetic material manufacturing	0.631–0.392	0.512
Special chemical product manufacturing	0.631–0.392	0.512
Daily chemical product manufacturing	0.631–0.392	0.512
Pharmaceutical manufacturing	0.631–0.392	0.512
Chemical fiber manufacturing	0.631–0.392	0.512
Cement, lime, and gypsum manufacturing	0.631–0.392	0.512
Cement and gypsum product manufacturing	0.631–0.392	0.512
Brick, stone, and other building material manufacturing	0.631–0.392	0.512
Glass and glassware manufacturing	0.631–0.392	0.512
Ceramic product manufacturing	0.631–0.392	0.512
Refractory product manufacturing	0.631–0.392	0.512
Graphite and other nonmetallic mineral product manufacturing	0.631–0.392	0.512
Nonferrous metal and alloy manufacturing	0.631–0.392	0.512
Boiler and prime mover manufacturing	0.631–0.392	0.512
Metalworking machinery manufacturing	0.631–0.392	0.512
Hoist-transportation machine manufacturing	0.631–0.392	0.512
Pumps, valves, compressors, and similar machinery manufacturing	0.631–0.392	0.512
Other common equipment manufacturing	0.631–0.392	0.512
Mining, metallurgy, and building equipment manufacturing	0.631–0.392	0.512
Chemical, wood, and nonmetal processing equipment manufacturing	0.631–0.392	0.512
Special machinery for agriculture, forestry, husbandry, and fishery manufacturing	0.631–0.392	0.512
Other specialized equipment manufacturing	0.631–0.392	0.512
Railway transport equipment manufacturing	0.631–0.392	0.512
Automobile manufacturing	0.631–0.392	0.512
Vessel and floating facility manufacturing	0.631–0.392	0.512
Other transportation equipment manufacturing	0.631–0.392	0.512
Motor manufacturing	0.631–0.392	0.512
Transmission and distribution and control equipment manufacturing	0.631–0.392	0.512
Wire, cable, fiber optic cable, and electrical equipment manufacturing	0.631–0.392	0.512
Household electric and nonelectric appliance manufacturing	0.631–0.392	0.512
Other electrical machinery and equipment manufacturing	0.631–0.392	0.512
Communication equipment manufacturing	0.631–0.392	0.512
Radar and radio equipment manufacturing	0.631–0.392	0.512
Electronic computer manufacturing	0.631–0.392	0.512
Electronic component manufacturing	0.631–0.392	0.512
Home audio-visual equipment manufacturing	0.631–0.392	0.512
Other electronic equipment manufacturing	0.631–0.392	0.512
Instrument manufacturing	0.631–0.392	0.512
Cultural and office machinery manufacturing	0.631–0.392	0.512
Craft and other product manufacturing	0.631–0.392	0.512
Production and supply of power and heat	2.323–1.569	1.946
Production and supply of gas	2.323–1.569	1.946
Production and supply of water	2.323–1.569	1.946
Construction	2.513–1.769	2.141
Railway transportation	2.993–1.914	2.454
Road transportation	2.993–1.914	2.454
City's public transportation	2.993–1.914	2.454
Water transportation	2.993–1.914	2.454
Air transportation	2.993–1.914	2.454
Pipeline transportation	2.993–1.914	2.454
Handling and other transport service	2.993–1.914	2.454
Warehousing	2.993–1.914	2.454
Wholesale and retail sale	0.44–0.15	0.295
Insurance	2.279–1.607	1.943
Resident service	0.655–0.312	0.484
Other service	0.655–0.312	0.484

Data source: Zou et al. [14].

**Table 3:** Results of economic effects (measuring unit: ten thousand RMB).

Industry	Direct service effect	Associated economic effect	Indirect economic effect	Complete economic effect
Agriculture	224274.860	32254.572	307400.052	415246.092
Forestry	45652.060	67369.404	68169.407	80815.325
Animal husbandry	157244.380	13094.821	180582.946	245308.922
Fishery	65774.200	2730.388	69725.861	80974.323
Service of agriculture, forestry, husbandry, and fishery	22176.230	36522.675	31415.316	34289.819
Coal mining and washing	/	29468.833	64986.176	106900.696
Oil and gas exploration	/	2002.814	59147.069	96862.943
Ferrous metal ore mining	/	3.147	15374.575	17790.070
Nonferrous metal mining	/	6192.810	27888.475	35079.747
Other nonmetal ore mining	/	180479.691	27467.146	31787.500
Grain grinding	/	40438.204	8231.043	22338.272
Feed processing	/	76422.147	29632.467	39312.748
Vegetable oil processing	/	7223.678	3069.876	11041.238
Sugar industry	/	354.072	11.208	86.284
Slaughtering and meat processing	/	4688.495	3631.551	16511.096
Aquatic product processing	/	886.262	1136.525	6718.374
Other food processing	/	603.799	1374.011	16120.633
Convenience food manufacturing	1294.285	188.289	1363.028	7520.514
Milk and dairy manufacturing	541.542	175.256	716.447	4223.064
Condiments and fermentation products manufacturing	1733.750	595.822	2805.411	5249.706
Other food manufacturing	4296.243	129.679	4683.159	25718.227
Alcohol and wine manufacturing	3949.839	742.590	6565.676	20779.630
Soft drink and refined tea processing	/	368.611	729.371	3786.235
Tobacco processing	/	90.931	205.572	14109.853
Cotton, chemical fiber textile and printing, and dyeing finishing	/	2263.104	11392.778	36670.132
Weaving, dyeing, and finishing	/	424.478	308.375	5435.661
Linen textile, silk spinning, and finishing	/	1519.773	2792.121	7674.866
Textile product manufacturing	2318.879	11662.786	7111.676	12948.707
Knitwear, weaving, and its product manufacturing	3286.149	1361.131	4670.416	7287.079
Textile wearing apparel, footware, and cap manufacturing	7537.976	18570.466	19011.769	43126.773
Leather, fur, feathers (fine hair), and its product manufacturing	/	1591.953	9140.069	30904.725
Timber, wood, bamboo, rattan, palm, and straw processing	/	51003.512	23097.462	38102.199
Furniture manufacturing	7927.270	13202.959	11774.138	20665.662
Paper and paper product manufacturing	/	3417.195	20861.898	45138.795
Printing and copying for recording medium	/	2752.207	4972.889	17553.695
Sport, cultural, and educational supply manufacturing	5552.497	12173.136	14409.494	22219.404
Oil and nuclear fuel processing	/	53732.201	45330.912	74672.808
Coking	/	134.923	30533.565	35546.660
Basic chemical raw material manufacturing	4313.508	7067.420	27771.611	37444.661
Fertilizer manufacturing	4776.596	113030.519	46324.485	60428.552
Pesticide manufacturing	3479.818	123788.558	30955.208	40371.197
Coating, printing ink, paint, and similar product manufacturing	2246.195	73507.519	12012.993	16516.688
Synthetic material manufacturing	4390.088	8384.865	20399.731	28660.573
Special chemical product manufacturing	5617.388	5959.214	32088.072	44742.483
Daily chemical product manufacturing	5380.403	3648.736	8484.226	20895.037
Pharmaceutical manufacturing	14668.068	4186.108	22027.422	29524.215
Chemical fiber manufacturing	5324.564	9295.692	13389.803	19345.855
Rubber product manufacturing	/	36257.879	16756.519	26013.082
Plastic product manufacturing	/	67906.036	19624.883	38499.650
Cement, lime, and gypsum manufacturing	8157.829	185923.581	40322.398	49618.238
Cement and gypsum product manufacturing	1465.631	230146.363	7133.046	7690.405
Brick, stone, and other building material manufacturing	934.492	144240.892	4230.565	7271.283
Glass and glassware manufacturing	3514.849	36133.173	16109.635	22741.921
Ceramic product manufacturing	1071.437	116648.089	3055.160	4717.192
Refractory product manufacturing	23.347	32815.366	108.039	121.212
Graphite and other nonmetallic mineral product manufacturing	1815.357	23832.294	5177.474	6063.783
Ironmaking	/	123.489	2086.748	2427.965
Steelmaking	/	92.345	9490.169	11074.714
Steel rolling processing	/	253850.953	192794.942	223013.850
Ferroalloy smelting	/	915.104	8348.284	9723.447
Nonferrous metal and alloy manufacturing	15380.644	8219.925	59352.409	72456.541
Nonferrous metal rolling process	/	16988.377	35441.068	45998.841
Metal product manufacturing	/	46209.909	18022.247	31824.760
Boiler and prime mover manufacturing	1585.843	15390.963	5216.272	6956.593
Metalworking machinery manufacturing	1317.524	8476.230	4777.305	6708.909
Hoist-transportation machine manufacturing	751.089	560.301	1799.648	2400.930
Pumps, valves, compressors, and similar machinery manufacturing	747.080	18494.049	3112.927	4196.952
Other common equipment manufacturing	2549.709	33004.312	15292.951	19117.583
Mining, metallurgy, and building equipment manufacturing	1225.585	34734.644	5878.842	7334.650
Chemical, wood, and nonmetal processing equipment manufacturing	153.743	1372.453	594.162	828.217
Special machinery for agriculture, forestry, husbandry, and fishery manufacturing	542.986	86045.197	5151.476	6327.882
Other specialized equipment manufacturing	10897.859	7327.467	47018.932	66889.403
Railway transport equipment manufacturing	186.199	18862.441	3037.940	4247.180
Automobile manufacturing	6337.792	31957.175	31643.484	46873.349
Vessel and floating facility manufacturing	2552.346	5227.158	4394.514	5606.492
Other transportation equipment manufacturing	2430.771	535.248	2891.412	6278.044
Motor manufacturing	2773.806	27926.379	13458.015	17086.261
Transmission, distribution, and control equipment manufacturing	1124.588	54310.877	6471.885	8334.190
Wire, cable, fiber optic cable, and electrical equipment manufacturing	4094.003	114155.168	16208.657	21175.912
Household electric and nonelectric appliance manufacturing	4924.380	23225.400	7882.982	17166.417
Other electrical machinery and equipment manufacturing	10017.357	14318.681	21735.315	27076.277
Communication equipment manufacturing	2352.241	2951.962	4628.916	10437.479
Radar and radio equipment manufacturing	181.233	1602.207	281.370	326.689
Electronic computer manufacturing	1193.221	17732.838	4494.400	9978.567
Electronic component manufacturing	5151.759	6807.819	17136.031	27029.060
Home audio-visual equipment manufacturing	3591.788	1461.821	4379.862	20785.160
Other electronic equipment manufacturing	871.793	6197.221	1338.956	1758.516
Instrument manufacturing	3068.764	9233.189	12540.223	17682.669
Cultural and office machinery manufacturing	603.192	48454.565	2153.542	3659.179
Craft and other product manufacturing	3195.546	66736.291	11051.372	16906.321
Scrap waste	/	236.007	330.902	416.647
Production and supply of power and heat	86655.828	26555.778	232416.262	384403.028
Production and supply of gas	830.203	2633.266	1794.680	3742.844
Production and supply of water	7733.696	37911.991	12831.484	21718.429
Construction	384517.177	783.857	398017.605	411185.018
Railway transportation	44262.798	34882.827	139320.673	198323.547
Road transportation	100498.662	54551.412	180734.309	225390.320
City's public transportation	8640.534	20198.200	12884.220	16904.497
Water transportation	2287.128	14447.927	4474.668	7291.585
Air transportation	3791.430	19799.388	7387.494	11854.599
Pipeline transportation	/	0.000	0.000	0.000
Handling and other transport service	1992.648	10219.544	3805.300	5947.536
Warehousing	2736.210	9417.221	3598.809	4164.856
The postal service	/	22052.628	2228.910	4591.521
Telecommunication and information transmission service	/	12765.332	12776.421	39300.447
Computer service	/	2211.991	1412.032	2692.011
Software	/	517.298	70.752	184.895
Wholesale and retail sale	28363.365	33103.282	171367.334	286838.534
Accommodation	/	20236.829	7528.296	16449.092
Catering	/	22786.509	25574.979	49250.285
Banking, security, and other financial activity	/	33651.299	46885.264	82681.399
Insurance	/	38477.101	5864.920	11096.667
Real estate	/	6902.654	18166.860	78204.631
Leasing	/	118605.765	835.384	2422.092
Business service	/	15666.721	6283.722	10785.177
Tourism	/	74.551	128.804	3505.748
Research and experimental development	/	117186.760	1598.279	1857.017
Professional and technical service	/	136052.891	4321.458	5614.004
Science and technology exchange and promotion service	/	20825.614	803.687	1126.383
Geological exploration	/	0.000	0.000	0.000
Water management	/	115937.570	2979.632	4471.497
Environmental management	/	9469.365	1190.529	2282.958
Public facilities management	/	523.006	110.174	1655.620
Resident service	2045.384	13369.132	5267.168	13544.352
Other service	3366.220	26964.684	20683.657	33635.381
Education	/	2670.063	8415.407	33121.785
Health	/	3211.955	15980.637	33957.909
Social security	/	7518.737	82.898	106.042
Social welfare	/	4109.687	92.954	114.111
Press and publication	/	30820.604	2182.198	4817.494
Radio, television, film, and motion picture	/	158.709	148.309	1230.560
Culture and art	/	23.727	0.957	553.198
Sport	/	0.000	0.000	109.717
Entertainment	/	18301.346	4832.876	8490.062
Public management and social organization	/	3246.067	5919.578	7626.480

Total	1388263.853	3952190.571	3399128.942	5010533.474
